# The impact of the COVID‐19 pandemic on melanoma diagnoses

**DOI:** 10.1002/jvc2.15

**Published:** 2022-03-13

**Authors:** Patrick Weltler, Klemens Rappersberger, Peter Filzmoser, Igor Vujic

**Affiliations:** ^1^ Faculty of Medicine Sigmund Freud Private University Vienna Austria; ^2^ Department of Dermatology Klinik Landstraße Vienna Austria; ^3^ Institute of Pathology “In the Centre,” St. Poelten Austria; ^4^ Institute of Statistics and Mathematical Methods in Economics University of Technology Vienna Austria

**Keywords:** Breslow thickness, COVID‐19, lockdown, melanoma

## Abstract

**Introduction:**

We investigated whether governmental measures and lockdowns during the COVID‐19 pandemic had an impact on the number and histopathologic stages of melanoma.

**Methods:**

The number and thickness (Breslow) of all diagnosed melanomas per day, month, or period at the ‘Institute for Pathology in the Centre’ in 2019 and 2020 were compared. For 2020, we defined four time periods: Period 1: 1 January–15 March; Period 2: 16 March–15 May (Lockdown 1); Period 3: 16 May–2 November; Period 4: 3 November–7 December (Lockdown 2).

**Results:**

We found similar melanoma numbers in 2019 (577) and 2020 (608). The mean number of diagnoses per day during Lockdown 1 (Period 2) was significantly lower (0.87 melanomas/day; *p* = 0.005) when compared to the respective time periods in 2019 and to the other three periods in 2020 (Period 1: 1.65 melanomas/day, Period 3: 1.77 melanomas/day, and Period 4: 2.49 melanomas/day). Tumour thickness in July 2020 (1.9 mm) was significantly higher (*p* = 0.02) than in July 2019 (1.1 mm).

**Discussion:**

The significant lower number of histopathologic diagnoses of melanoma during ‘Lockdown 1’ may be explained by postponed or missed patient consultations. This assumption is supported by the demonstration of a higher tumour thickness in July and August 2020, compared to 2019.

## INTRODUCTION

Melanoma is potentially lethal skin cancer. Breslow thickness and ulceration are the most important prognostic factors in primary lesions.^1^ It is beyond any dispute that an early diagnosis and subsequent surgical excision are key for melanoma survival.^2^

During the COVID‐19 pandemic, some media claimed that legal regulations (‘lockdowns’) had led to a delay in medical visits. If this were true, an increased number of melanomas following a ‘lockdown’ would have been diagnosed, and the tumours would have shown a greater Breslow thickness.^3^, ^4^, ^5^, ^6^ To check this hypothesis, we investigated the impact of ‘lockdowns’ on the diagnosis of melanoma in Austria.

## MATERIALS AND METHODS

After obtaining ethical approval (Num. 311 ‐ 2021) we collected histopathological data on the number and Breslow thickness of all primary melanomas diagnosed at the pathological institute ‘Pathologie im Zentrum’, in St.Pölten, Austria, in the period from 1 January 2019 to 31 December 2020. Next, we compared the number of melanomas and their median Breslow thickness for each month in 2019 with the respective values in 2020. Furthermore, we divided 2020 into four specific periods: Period 1 (‘prelockdown’), 1 January 2020 to 15 March 2020; Period 2 (‘first lockdown’), 16 March 2020 to 15 May 2020; Period 3 (‘between lockdowns’), 16 May 2020 to 22 November 2020; and Period 4 (‘second lockdown’), 3 November 2020 to 7 July 2020, which we compared to each other and to their respective periods in 2019 in terms of the number of melanomas and their Breslow thickness. For our calculations, we used the statistics programme ‘R’.^7^

## RESULTS

**Figure 1:**
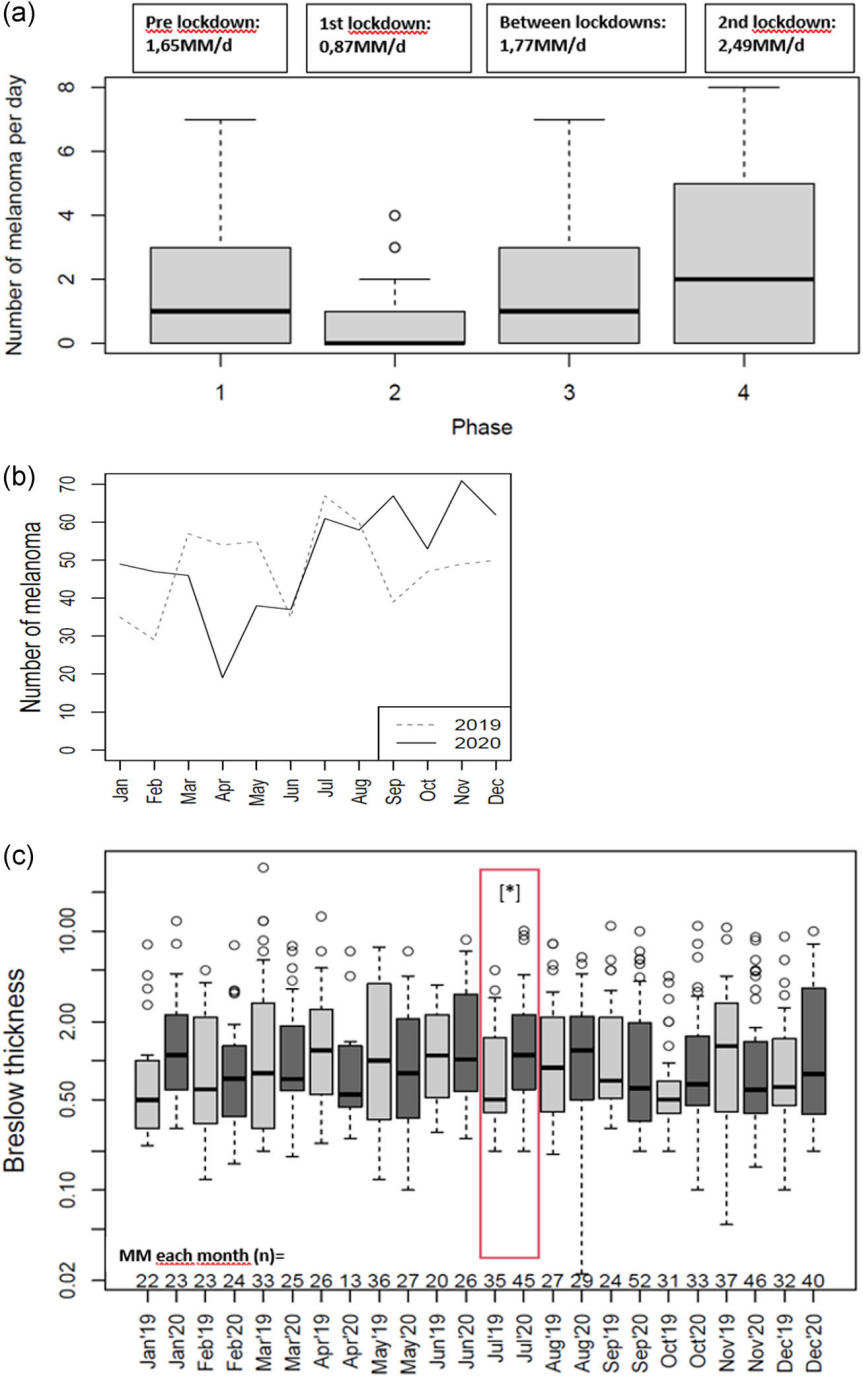
(a) Boxplot comparing the mean melanoma diagnoses per day in four phases of 2020: prelockdown, first Lockdown, in‐between the lockdowns and second lockdown. The distribution of melanoma diagnoses per day differs in the four periods in 2020 (Kruskal–Wallis test; *p* = 0.005). (b) Number of diagnosed melanomas per month (including in situ tumours) in each month of 2019 compared to 2020. (c) Boxplot comparing median Breslow thickness of diagnosed invasive melanomas (excluding the in situ tumours) for each month 2019–2020. Significant differences were found for July (Kolmogorov–Smirnow test; *p = 0.02)

We analysed data of 1185 primary melanomas, of which 577 and 608 were diagnosed in 2019 and 2020, respectively. Descriptive statistics and data on the average number of melanomas diagnosed per day in each given time period (the number of melanomas in each period divided by the number of equivalent days) and the mean Breslow thickness are shown in Table 1. The distribution of diagnoses of melanoma per day in the four periods in 2020 was significantly different (Kruskal–Wallis test; *p* = 0.005) with the lowest number of melanomas diagnosed per day during the first lockdown (Figure 1a). Melanoma frequencies per month in Years 2019 and 2020 were also different, showing a decline in diagnosis during the first lockdown period (Figure 1b). This was also confirmed by a Wald–Wolfowitz run test, where differences between the same months in the 2 years show a systematic pattern (*p* = 0.01).

**Table 1:** Melanoma diagnoses in 2019 and 2020: absolute counts, melanomas per day, patient‐age, and Breslow thickness for all four periods

Time period	Number of diagnosed melanomas (% of total per year)	Number of diagnosed melanomas (female) (% of the total in this period)	Average number of melanomas per day in this period	Median age	Mean Breslow (Median)
2019	577	273	1.58	69	1.73 (0.7)
1 Jan–15 Mar	90 (15.6)	44 (48.9)	1.22	74	1.65 (0.67)
16 Mar–15 May	114 (19.8)	58 (50.9)	1.87	69	2.53 (1.10)
16 May–2 Nov	274 (47.5)	128 (46.7)	1.6	67	1.44 (0.60)
3 Nov–7 Dec	62 (10.7)	25 (40.3)	1.77	69	1.80 (1.02)
2020	608	266	1.66	66	1.77 (0.8)
1 Jan–15 Mar (prelockdown)	124 (20.4)	53 (44.4)	1.65	62.5	1.90 (0.90)
16 Mar–15 May (first lockdown)	53 (8.7)	23 (43.4)	0.87	58	1.44 (0.62)
16 May–2 Nov (in‐between lockdown)	302 (49.7)	131 (43.4)	1.77	71	1.74 (0.83)
3 Nov–7 Dec (second lockdown)	87 (14.3)	44 (50.6)	2.49	63.5	1.64 (0.65)

Regarding mean Breslow thickness (Figure 1c), there were no significant differences between 2019 and 2020. However, there was a difference when July 2019 and July 2020 (period after the first lockdown) were compared (1.1 mm in July 2019 vs. 1.9 mm in July 2020; Kolmogorow–Smirnow test, *p* = 0.02).

## DISCUSSION

Ours and other studies show that fewer melanomas were diagnosed during the first lockdown period, probably due to postponed or missed consultations induced by government regulations.^6^ The increase in melanoma diagnoses seen during the second lockdown (first lockdown, 0.87 melanomas per day vs. second lockdown, 2.49 melanomas per day), may be explained by the fact that government measures and their widespread consequences may have become less effective compared to the first lockdown, probably because of decreased awareness or decreased fear of the population later in 2020. The increase in melanoma diagnoses during the second lockdown probably reflects delayed medical checkups during the first lockdown.

According to the biological behaviour of melanoma, one would expect an increase in Breslow thickness after the first lockdown, due to delayed diagnoses and excisions. Surprisingly, in our study cohort, the Breslow thickness did not vary significantly after the first lockdown. However, in July 2020 we found statistical differences compared to July 2019. Similar data on the Breslow thickness was also reported by another Austrian group.^6^ However, these results differ from data reported in Italy, where lockdown phases were longer, and regulations were stricter than in Austria.^3^ A significant change in Breslow thickness could have been found if longer lockdowns had been implemented in Austria. Differences in health systems in different countries may also play a role; for example, most melanomas in Austria are excised in dermatologic practices that are easier to access than University hospitals or outpatient clinics. Despite we did not find significant differences in the Breslow thickness after lockdown periods, we think that melanoma diagnoses should not be delayed in the case of lockdowns in possible future pandemics.

## CONFLICTS OF INTEREST

The authors declare no conflicts of interest.

## FUNDING INFORMATION

This study was not funded.

## Data Availability

Author decides not to share data.
